# Mental Fatigue Monitoring Using a Wearable Transparent Eye Detection System

**DOI:** 10.3390/mi7020020

**Published:** 2016-01-26

**Authors:** Kota Sampei, Miho Ogawa, Carlos Cesar Cortes Torres, Munehiko Sato, Norihisa Miki

**Affiliations:** 1 Department of Mechanical Engineering, Keio University, 3-14-1 Hiyoshi, Kohoku-ku, Yokohama, Kanagawa 223-8522, Japan; kota.sampei@gmail.com (K.S); notuned.miho@gmail.com (M.O); carloscortes1103@gmail.com (C.C.C.T); 2 Media Lab, Massachusetts Institute of Technology, 77 Massachusetts Ave., Cambridge, MA 02139, USA; munehiko@acm.org; 3 JST PRESTO, 7 Gobancho, Chiyoda-ku, Tokyo 102-0076, Japan

**Keywords:** eye, wearable, micro, microelectromechanical systems, dye sensitized photovoltaic device, sensor, mental state, monitoring, fatigue

## Abstract

We propose mental fatigue measurement using a wearable eye detection system. The system is capable of acquiring movement of the pupil and blinking from the light reflected from the eye. The reflection is detected by dye-sensitized photovoltaic cells. Since these cells are patterned onto the eyeglass and do not require external input power, the system is notable for its lightweight and low power consumption and can be combined with other wearable devices, such as a head mounted display. We performed experiments to correlate information obtained by the eye detection system with the mental fatigue of the user. Since it is quite difficult to evaluate mental fatigue objectively and quantitatively, we assumed that the National Aeronautics and Space Administration Task Load Index (NASA-TLX) had a strong correlation with te mental fatigue. While a subject was requested to conduct calculation tasks, the eye detection system collected his/her information that included position, velocity and total movement of the eye, and amount and frequency of blinking. Multiple regression analyses revealed the correlation between NASA-TLX and the information obtained for 3 out of 5 subjects.

## 1. Introduction

Assessment of the physical and mental states of the workers is of great benefit to enhance the work efficiency and secure the safety, amongst which we consider that the mental fatigue or stress that we experience is one of the most important factors. Subjective ratings are widely used to describe the physical and mental states and to assess the workload of tasks. National Aeronautics and Space Administration Task Load Index (NASA-TLX) was developed to quantitatively estimate the workload [1]. However, this method requires both *a priori* and *a posteriori* ratings by the subjects. To quantify the stress level objectively, biomarkers, such as salivary amylase, that are contained in the saliva have been used. However, it takes time for the biomarkers to respond to the stress that the subject experiences and also to be detected [2,3,4]. Electroencephalograms (EEGs), or brain waves, reflect the brain activity and are used to visualize the mental state [5,6]. EEG recording system is composed of electrodes, an amplifier, and a logger that are all wired, which hinders the subjects’ activities. In addition, conventional wet EEG electrodes require pretreatment of the skin. Dry electrodes that do not need skin pretreatment or conductive gel are currently studied [7,8], which may allow EEGs to be used for the mental fatigue monitoring with a miniaturized measurement system. Heart rates, in particular, the high frequency and low frequency activities, are known to correlate with the fatigue level of the subjects [9,10,11]. Thanks to the relatively ease in measurement and analysis, assessment of the fatigue based on the heart rates is now widely used. 

**Figure 1:**
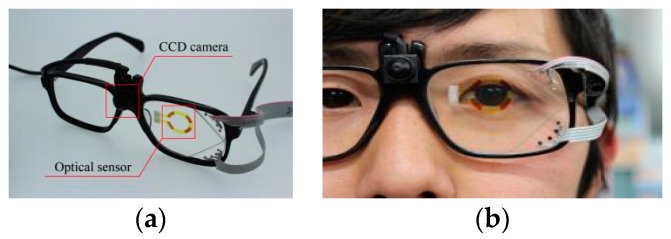
(**a**) See-through-type wearable eye-tracking system. The colored parts are dye-sensitized photovoltaic cells that detect the light reflected from the eye and deduce the pupil position and eye blink. A charge-coupled device (CCD) camera is used to detect the line of sight of the user. When we only need to detect the movement of the eye and eye blinking, the CCD camera is not used and no external power is necessary. (**b**) The eye-tracking system in use. Since the distance between the sensor cells and the eye is a few centimeters, the user does not notice the cells and can see through them as if he/she were wearing sunglasses with the color of the dye.

In order to feedback the mental fatigue level to augment the work efficiency and maintain the safety, the assessment needs to be conducted real-time without disturbing the user’s activity. Therefore, the subjective rating and detection of biomarkers are not suitable for the purpose. In this paper, we report preliminary results to assess the mental fatigue by the movement of the eye that is acquired by a wearable device. We consider that the eyeglasses-type device is one of the most familiar and user-friendly wearable devices. It is often said that the eye is a window to the soul and it is true that we judge others’ physical and mental states from their appearances, especially around the eyes [12,13,14]. Wearable eye trackers are now commercially available, many of which use external cameras to obtain the limbus of the pupil or acquire a Purkinje image, which is the reflection of the near infrared light from the eye [15,16,17]. In order to use the eye movement to assess the user’s states, detection of the eye movement needs to be conducted without disturbing the subjects, where light weight is essential and low-power-consumption is preferable for long term monitoring and unnecessity of batteries. We have developed a see-through-type eye-tracking system using photovoltaic cells as transparent optical sensors on eyeglasses [18,19,20]. Dye-sensitized photovoltaic cells, which have been studied as transparent solar cells [21], are microfabricated on the eyeglasses, as shown in Figure 1. To deduce the pupil position, the four cells detect the reflection of light from the eye, since the reflection is weak over the pupil and strong over the white parts of the eye. Due to the gap between the sensor and the eye, it was found that the output voltage of the cells is approximately proportional to the horizontal distance from the pupil center. This system detects the rotational angle of the pupil with an accuracy of 1.5° [19]. In addition, the system successfully detects eye blinks. The main advantage of this system is that it does not need any cameras, is very lightweight (sensors < 200 mg, total weight < 30 g) and does not require an external power source. 

The ultimate goal of this work is to extract the parameters of the eye movement that has strong correlation with the mental fatigue. This paper highlights the procedure and experiments, where the microfabrication-enabling wearable eye detection system play a critical role. We assumed that workload deduced by the NASA-TLX represented the mental fatigue of the subjects. The subjects were requested to complete tasks, which were mental calculations, while the pupil detection system collected information from them. As the parameters, we selected the positions and movements of eyes, and the frequency of eye blinks. The correlation between the workload and the information collected by the pupil detection system was later analyzed. 

## 2. Experimental Section

### 2.1. Concept of the Wearable Eye Detection System

**Figure 2:**
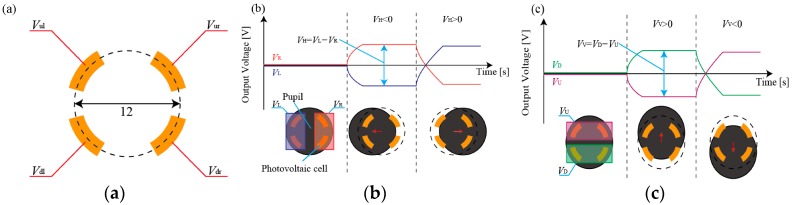
(**a**) Four photovoltaic cells patterned onto the eyeglasses. (**b**) The horizontal position of the pupil can be deduced from the difference between *V*_L_ and *V*_R_, which are the average of the two left cells (*V*_ul_ and *V*_dl_) and the two right cells (*V*_ur_ and *V*_dr_). (**c**) The vertical position of the pupil can be deduced from the difference between *V*_U_ and *V*_D_, which are the averages of the two upper cells (*V*_ul_ and *V*_ur_) and the two bottom cells (*V*_dl_ and *V*_dr_).

Figure 2 shows the process to deduce the pupil position from the output of the four photovoltaic cells. The horizontal position of the pupil can be deduced from the difference between *V*_L_ and *V*_R_, which are the average of the two left cells (*V*_ul_ and *V*_dl_) and the two right cells (*V*_ur_ and *V*_dr_). The vertical position of the pupil can be deduced from the difference between *V*_U_ and *V*_D_, which are the averages of the two upper cells (*V*_ul_ and *V*_ur_) and the two bottom cells (*V*_dl_ and *V*_dr_). It was reported that the *V*_H_, which is the difference between *V*_L_ and *V*_R_, and *V*_V_, the difference between *V*_U_ and *V*_D_, have linear relationship with respect to the horizontal and vertical view angle of a subject, respectively, which is because of the gap of approximately 10 mm between the sensor and the eye [19]. We calibrated the system as follows; The subject was requested to look at target points 1 m in front of him. The relationship between the *V*_H_ and the horizontal view angle and *V*_V_ and the vertical view angle was deduced. Note that the trend was different between when the view angle is positive and negative due to the asymmetricity of the eye. The calibration results for one subject are shown in Supplemental Materials (Figure S1). The eye detection system has four cells to detect the pupil position. The small number of cells is beneficial to increase the yield by reducing the difficulty of microfabrication. 

**Figure 3:**
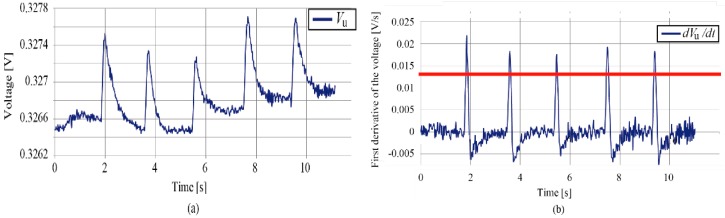
(**a**) Output voltage and (**b**) the first derivative when the subject blinks. The system clearly detects the eye blinks. The red line in Figure 3b represents the threshold value for blink detection.

In addition, the cells can detect eye blinks [19]. As shown in Figure 3, the light reflected from the eye lid was detected. In this paper, we requested the subjects to blink 5 times and then deduce the average of the first derivative with respect to time (Figure 3b), which was conducted prior to the tasks. Then, we set the threshold as the 70% of the average to detect the eye blinks, which was 0.013 V/s in this case. It was reported that the typical eye blink has a period of 100 to 200 ms [22]. As shown in Figure 3b, the first derivative dropped below the threshold in approximately 300 ms. Therefore, we consider that the device can identify the eye blinks reliably. In our experiments, we did not observe blinks at such a high frequency that they were not detected by our system. In the calibration experiments, the output of the sensors at the blinks did not vary much. However, at the normal blinks, *i.e.*, when the subjects were not requested to make blinks, the output varied, which was because the output was influenced by the position and movement of the eye. However, the blinks can be detected by the developed system. The first derivative of the voltage when the subject made normal blinks is shown in Supplemental Materials (Figure S2).

### 2.2. Experimental Protocol

#### 2.2.1. NASA-TLX

The experiments were approved by the bioethics board of the faculty of science and technology, Keio University. Five subjects, who were A (male, 25 years old, naked eye), B (male, 21, contact lens), C (male, 24, contact lens), D (female, 23, contact lens), and E (female, 23, naked eye), took part in the experiments. We presumed that the mental fatigue or mental stress of the subjects who experienced tasks, which are mental calculations in this work, can be represented by the workload of the task. We used NASA-TLX to deduce the workload. First, we explained the subjects the six defined sources of workload; mental demand (*MD*), physical demand (*PD*), temporal demand (*TD*), performance (*OP*), effort (*EF*), and frustration (*FR*). The instruction was made in Japanese, which is their native language. Then, the subjects were requested to compare the six defined sources of workload in a pairwise comparison method: Weights a, b, c, d, e and f were assigned to each of the six workload sources from the pairwise comparison method. The weights were integers ranging from 0 to 5, and their sum was 15. After the subjects completed the requested tasks, they were asked to evaluate the six factors on a 0 to 100 scale and then the workload was derived from a weighted average of the ratings of these six factors, as expressed in Equation (1).

(1) $$workload=\frac{a\;MD+b\;PD+c\;TD+d\;OP+e\;EF+f\;FR}{15}$$

#### 2.2.2. Tasks and Measurement

**Figure 4:**
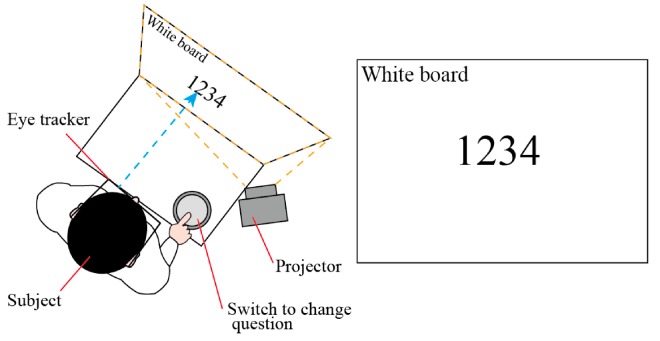
Experimental setup. The subject wears the eye tracker. A number with 3 or 4 digits is shown by the projector on the white board located 1 m in front of the subject. The switch to change the question is placed on the desk.

First, calibration experiments for the eye-tracking system were conducted on each subject. Then, the subjects were requested to perform mental calculations; for example, they answered the remainder when numbers with 3 or 4 digits were divided by 7. The number was shown on a white board 1 m in front of them and then replaced by the next problem when the subject pressed a switch, as shown in Figure 4. The subjects were requested to conduct the mental calculations for 8 min. They were asked to answer the questions as correctly as possible. The information on the eye positions and movements and the blinks was acquired during the last minute of the calculation tasks, *i.e.*, from 7 to 8 min. By acquiring the data only during the last minute, the amount of data to be analyzed were reduced while the subjects were supposed to have the largest fatigue by the task. In the following 2 min, *i.e.*, from 8 to 10 min, the subjects rated the factors (*MD*, *PD*, *TD*, *OP*, *EF*, *FR*) to deduce the workload on a scale of 1 to 100. This procedure that consists of the calculation task for 8 min and parameter rating for 2 min was iterated in sequence six times, and so the tests took a total of 60 min for each subject. 

#### 2.2.3. Analysis

The ultimate goal of this work is to find one or a few parameters that are most correlated with mental fatigue, or workload. The parameters should be monitored by the wearable eye detection system on real time. The candidate parameters we selected here as the first step were the position of the eye (upper/bottom, right/left, upper-right/upper-left/bottom-right/bottom-left), motion of the eye, number of blinks, and number of multiple blinks, which represented how often the subject blinked more than twice a second. These parameters were deduced from the information that was acquired by the eye detection system. Other than the information acquired by the eye detection system, the number of the tasks (1 to 6) was also investigated. We conducted stepwise regression using IBM SPSS Statistics (IBM Corp., Armonk, NY, USA) to find which parameters have correlation with the workload of the six tasks. 

## 3. Results and Discussion

### 3.1. Workload of the Requested Tasks

Based on the comparison among the six factors (*MD*, *PD*, *TD*, *OP*, *EF*, *FR*) prior to the tasks, we formulated equations to calculate the workload for all five subjects (A–E), as shown in Equations (2–6), respectively. The subscripts represent the subject.

(2) $$workload_{A}=\frac{5MD_{A}+TD_{A}+4OP_{A}+3EF_{A}+2FR_{A}}{15}$$

(3) $$workload_{B}=\frac{4MD_{B}+PD_{B}+3TD_{B}+2EF_{B}+5FR_{B}}{15}$$

(4) $$workload_{C}=\frac{3MD_{C}+4TD_{C}+4OP_{C}+2EF_{C}+2FR_{C}}{15}$$

(5) $$workload_{D}=\frac{4MD_{D}+2PD_{D}+TD_{D}+5OP_{D}+EF_{D}}{15}$$

(6) $$workload_{E}=\frac{MD_{E}+2PD_{E}+5TD_{E}+3EF_{E}+4FR_{E}}{15}$$

Based on these equations and the rating of the six factors after the tasks, the workloads were calculated and are listed in Table 1. The task was mental calculation and iterated 6 times. The workloads did not increase monotonically with the number of iterations since the subjects became familiar with the tasks during the experiments. For all the subjects, the workload of the 6th task was the largest.

**Table 1:** Workload of the tasks for subjects A–E.

The Number of the Tasks	Workload				
	A	B	C	D	E
1	60.0	63.5	26.3	63.5	46.3
2	59.0	58.3	33.3	58.3	62.3
3	66.0	61.7	20.7	61.7	57.1
4	72.0	60.7	55.3	60.7	66.0
5	86.3	54.7	52.3	54.7	75.0
6	93.0	76.9	56.3	76.9	85.7

### 3.2. Information Acquired by the Eye-Tracking System and the Correlation with the Workload

As we described in Section 2.2.3, we selected the positions of the eye in the upper and bottom side, left and right, upper-left, upper-right, bottom-left, and bottom-right, the amount of movement of the eye, the number of blinks, the number of multiple blinks (the subject blinked more than twice a second), and the number of the tasks (Task#). The values of these parameters along with the workload in the case of subject A is shown in Supplemental Materials (Table S1).

We conducted stepwise regression using IBM SPSS Statistics to investigate the correlation between the parameters and the workload of the six tasks for subjects A–E. The deduced correlation between the workload and the parameters for each subject is described in Equations (7)–(11), respectively. The subscripts represent the subjects.

(7) $$workload_{A}=6.54+6.58\times \left(\text{Task}{\#}_{A}\right)+19.8\times \left({\text{BottomLeft}}_{A}\right)-0.006\times \left({\text{EyeMovement}}_{A}\right)$$

(8) $$workload_{B}=75.5-0.546\times \left({\text{Blinks}}_{B}\right)-2.13\times \left({\text{MultipleBlinks}}_{B}\right)$$

(9) $$workload_{C}=34.1+6.13\times \left(\text{Task}{\#}_{C}\right)$$

(10) No correlation

(11) $$workload_{E}=37.1+6.65\times \left(\text{Task}{\#}_{E}\right)+0.951\times \left({\text{MultipleBlinks}}_{E}\right)$$

In the cases of subjects C and D, no correlation was found between the workload and the eye information that were selected and investigated in this work. 

We closely looked into the cases of subjects A, B and E. In the case of A, the determination coefficient was found to be 0.999. All three selected parameters had a significance probability less than 5%. Multicollinearity was not found. 

In the case of B, multicollinearity was found. Therefore, we removed the number of eye blinks from the equation and deduced the regression model as follows:

(12) $$workload_{B}=75.5-1.76\times \left({\text{MultipleBlinks}}_{B}\right)$$

The determination coefficient was 0.933 and the significance probability was deduced to be 0.001 in *F*-tests.

In the case of E, no multicollinearity was found and the determination coefficient was 0.986. All the parameters had a significance probability less than 5%.

From these results, we can say that the workload can be estimated by the information acquired by the developed eye detection system for the three subjects (A, B and E) out of five. The number of multiple blinks was influential in the cases of B and E, but not in the case of A. 

## 4. Conclusions

We conducted experiments to detect the mental fatigue level of five subjects using the wearable eye detection system. We assumed that mental fatigue could be represented by the workload deduced by NASA-TLX. We consider that the proposed eye detection system is effective in mental fatigue monitoring during the tasks, since it is lightweight and does not have any external cameras that block the eye sight or produce mental stress. From the experiments with five subjects, three subjects showed correlation between the workload (mental fatigue) and the information acquired by the eye detection system. The results verified the feasibility that the micro technology-enabled eye-tracking system can monitor the fatigue level of participants performing the tasks. However, for the other two subjects, the correlation was not found. Further experiments need to be conducted to find the specific eye information that strongly correlates to the mental fatigue level for all the subjects.

## Supplementary Materials

The following are available online at http://www.mdpi.com/2072-666X/7/2/20/s1. Figure S1: Relations between the viewing angle and the output voltage (**a**) *V*_H_ and (**b**) *V*_V_ for one subject. Figure S2: First derivative of the output voltage of the sensor when the subjects blinked naturally. Table S1: Acquired information for subject A.
