# Supplementary material for: Mental Fatigue Monitoring Using a Wearable Transparent Eye Detection System
Source: Micromachines (Basel). 2016 Jan 26;7(2):20. doi: 10.3390/mi7020020 (PMC6189833; doi:10.3390/mi7020020)
Supplement: Supplementary file 1 [file micromachines-07-00020-s001.pdf]

Supplementary Materials: Mental Fatigue Monitoring Using a Wearable Transparent Eye Detection System

Kota Sampei, Miho Ogawa, Carlos Cesar Cortes Torres, Munehiko Sato and Norihisa Miki

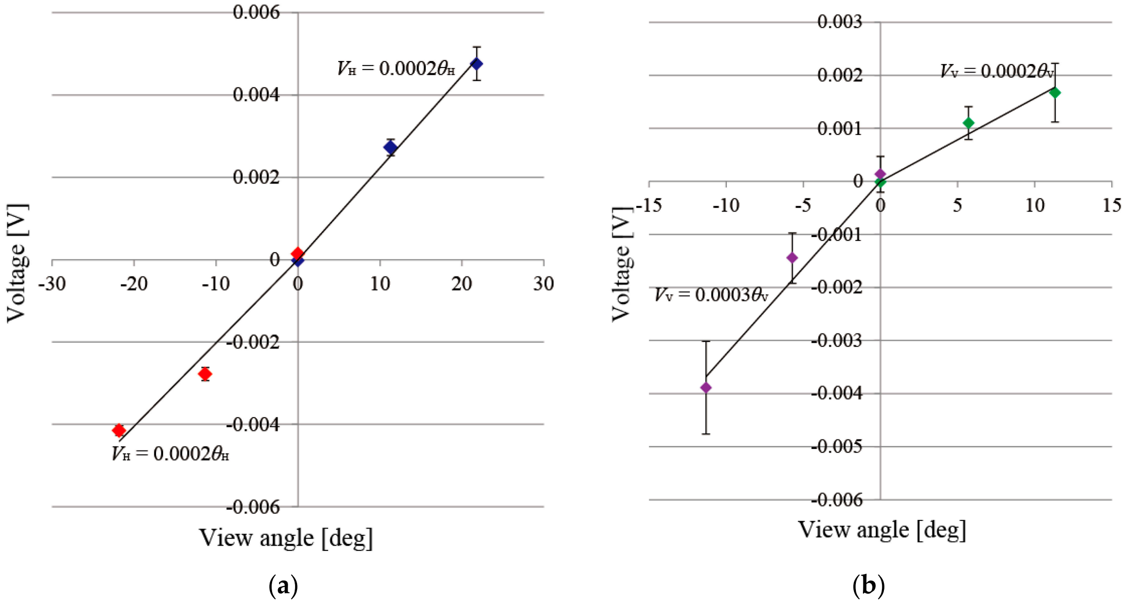

Figure S1. Relations between the viewing angle and the output voltage (**a**) V_H and (**b**) V_V for one subject. These relations are obtained to calibrate the system prior to the monitoring experiments.

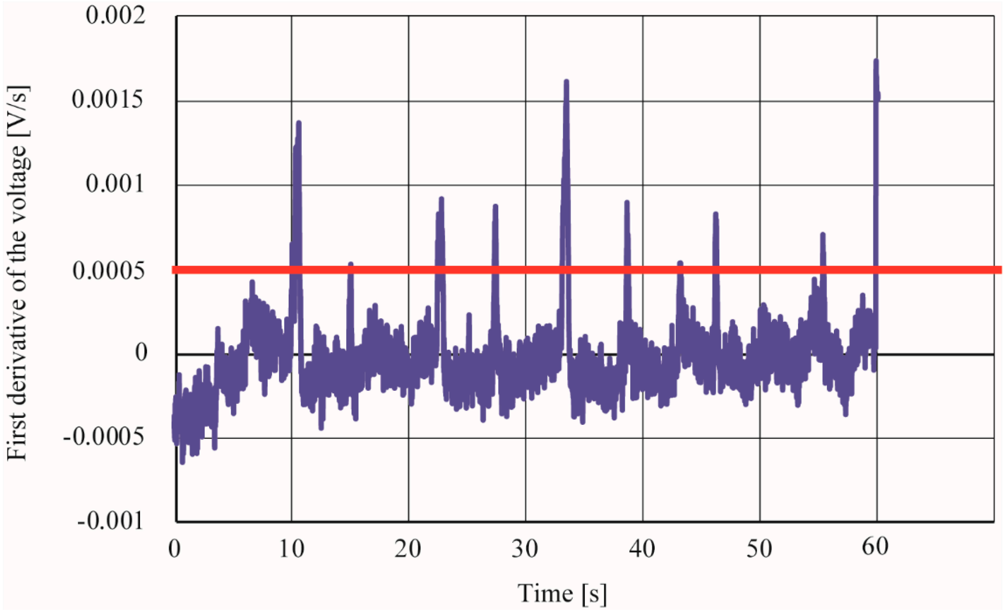

Figure S2. First derivative of the output voltage of the sensor when the subjects blinked naturally. The blinks were detected for 60 s while the subject was not requested any tasks.

Table S1. Acquired information for subject A.

The Number of the Tasks	Upper	Bottom	Left	Right	Upper-Left	Upper-Right	Bottom-Left	Bottom-Right	Eye Movement (°)	No. of Blinks	No. of Multiple Blinks
1	0.39	0.61	0.10	0.90	0.04	0.35	0.07	0.54	2080	45	14
2	0.64	0.36	0.63	0.37	0.44	0.20	0.19	0.17	3692	34	12
3	0.04	0.96	0.05	0.95	0.00	0.04	0.04	0.92	3053	36	7
4	0.99	0.01	0.03	0.97	0.02	0.97	0.00	0.00	3130	45	12
5	0.40	0.60	0.00	1.00	0.00	0.39	0.00	0.60	1921	34	6
6	0.93	0.07	0.85	0.15	0.80	0.14	0.06	0.01	1998	31	5
